# The DREEM, part 2: psychometric properties in an osteopathic student population

**DOI:** 10.1186/1472-6920-14-100

**Published:** 2014-05-20

**Authors:** Brett Vaughan, Jane Mulcahy, Patrick McLaughlin

**Affiliations:** 1 Discipline of Osteopathic Medicine, College of Health & Biomedicine, Victoria University, Melbourne, Australia; 2 Institute of Sport, Exercise and Active Living, Victoria University, Melbourne, Australia

## Abstract

**Background:**

The Dundee Ready Educational Environment Measure (DREEM) is widely used to assess the educational environment in health professional education programs. A number of authors have identified issues with the psychometric properties of the DREEM. Part 1 of this series of papers presented the quantitative data obtained from the DREEM in the context of an Australian osteopathy program. The present study used both classical test theory and item response theory to investigate the DREEM psychometric properties in an osteopathy student population.

**Methods:**

Students in the osteopathy program at Victoria University (Melbourne, Australia) were invited to complete the DREEM and a demographic questionnaire at the end of the 2013 teaching year (October 2013). Data were analysed using both classical test theory (confirmatory factor analysis) and item response theory (Rasch analysis).

**Results:**

Confirmatory factor analysis did not demonstrate model fit for the original 5-factor DREEM subscale structure. Rasch analysis failed to identify a unidimensional model fit for the 50-item scale, however model fit was achieved for each of the 5 subscales independently. A 12-item version of the DREEM was developed that demonstrated good fit to the Rasch model, however, there may be an issue with the targeting of this scale given the mean item-person location being greater than 1.

**Conclusions:**

Given that the full 50-item scale is not unidimensional; those using the DREEM should avoid calculating a total score for the scale. The 12-item ‘short-form’ of the DREEM warrants further investigation as does the subscale structure. To confirm the reliability of the DREEM, as a measure to evaluate the appropriateness of the educational environment of health professionals, further work is required to establish the psychometric properties of the DREEM, with a range of student populations.

## Background

The educational environment can influence a student’s study habits and assessment results. The Dundee Ready Educational Environment Measure (DREEM) was developed by Roff et al. [1] to measure the educational environment in health professional education programs. A large number of papers that explore student perceptions of their tertiary institution and outcomes of their educational experience report the use of the DREEM as an outcome measure; many of these are summarised in the paper by Miles et al. [2]. Additional studies using the DREEM have been published since this review [3-13], however, other than Hammond et al. [3] none investigated the psychometric properties of the measure. Most studies that used the DREEM have reported a variety of descriptive statistics for the scale items, subscales and the total DREEM score; internal consistency (Cronbach’s alpha); and correlational statistics, investigating relationships between the DREEM total and subscale scores with characteristics such as age, gender, and program year level.

The DREEM scores are both subscale and total scores and interpreted according to the scale provided by McAleer & Roff [14]. In the development of the DREEM, the authors suggested that the items be structured around an a-priori 5 factor model. These factors are Perception of Teaching, Perception of Learning, Academic Self-perception, Perception of Atmosphere and Social Self-perception. A number of authors have investigated the factor structure of the DREEM and have failed to reproduce the 5-factor structure [3,15-17]. Hammond et al. [3] highlighted a number of issues with the psychometric properties of the DREEM. Their research indicated that in order to produce a fit for the original 5-factor model of the DREEM, 17 out of the 50 items had to be removed. Yusoff [18] produced five models of the DREEM using confirmatory factor analysis (CFA) (plus one one-factor model and an analysis of the original five-factor model) in a Malaysian medical student population. Model fit was only achieved with the original five-factor model with 17 items. An exploratory factor analysis conducted by Jakobsson et al. [17] revealed between five and nine factor solutions in their study of a Swedish version of the DREEM. These authors [3,17,18] have concluded that the internal consistency and construct validity of the measure is not stable, and that the model itself may need to be revised.

Part 1 of this two part series of papers presented an analysis of the DREEM using data gathered from students in the 5-year osteopathy program at Victoria University, Melbourne, Australia. The data presented in that paper scored and interpreted the data as five subscales and a total score, as suggested by Hammond et al. [3] and Miles et al. [2]. The aim of the present paper is to present the psychometric properties of the DREEM in this osteopathy student population, using a combination of classical test theory (CTT) and item response theory (IRT).

## Methods

### Study design

The design of the study has been described in detail in part 1 of this series of papers. The study was approved by the Victoria University Human Research Ethics Committee.

### Participants

Students enrolled in the Osteopathic Science subject in years 1–5 of the osteopathy program at Victoria University were invited to complete a demographic questionnaire and the DREEM.

### Data collection

Participants completed the DREEM and demographic questionnaire in their final Osteopathic Science class in semester 2, 2013 (October).

### Data analysis

**Figure 1:**
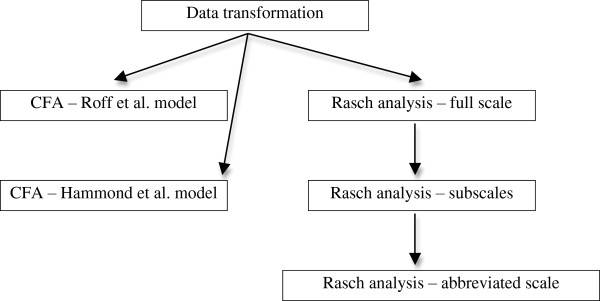
Data analysis process.

Data were entered into SPSS for Mac (IBM Corp, USA) for analysis. A flow diagram outlining the data analysis process is found at Figure 1. The data were transformed and a CFA was performed on the data set with the 5-factor structure identified by Roff et al. [19] and then on the 5-factor structure model proposed by Hammond et al. [3]. The SPSS data file was transferred to AMOS Version 21 (IBM Corp) for the CFA calculation using the Maximum Likelihood Estimation method. CFA investigates the fit of the data to the constructed model, and presents relationships between the data in the model and estimations of error. In the CFA a range of model fit statistics are generated to describe how the data fits the model being tested. Readers are encouraged to access Brown [20] and Schreiber et al. [21] who present further detail about the CFA process and the fit statistics. The data were not normally distributed - a bootstrapping procedure was applied for each of the two models, 1000 iterations of the data were generated. No changes to either of the models were made based on the results of the CFA.

Given the authors of the DREEM have recommended calculating a total score for the scale, a Rasch analysis is appropriate [22]. Rasch analysis provides a mathematical model of the data that is independent of the sample, rather than the sample dependent calculation used in classical test theory [23,24]. In this analysis, data are fitted to the Rasch mathematical model as closely as possible [23]. The data were converted in SPSS to an ASCII format and imported into the RUMM2030 (RUMM Laboratory, Australia) program for Rasch analysis, where the polytomous Partial Credit Model was used.

The RUMM2030 program produces three model fit statistics in order to determine the fit to the model. The first is an item-trait chi-square (χ^2^) statistic demonstrating the invariance across the trait being measured. A statistically significant Bonferonni-adjusted χ^2^ indicates misfit to the Rasch model. The other two statistics relate to the item-person interaction, where the data is transformed to approximate a *z* distribution. A fit to the Rasch model is indicated by a mean of 0 and a standard deviation (SD) of 1. Further, individual item and person statistics are presented as residuals and a χ^2^ statistic. Residual SD’s greater than ± 2.5 and/or significant Bonferroni-adjusted χ^2^ statistics indicate poor item fit, and residual SD’s greater than ± 2.5 indicate a poorly fitting person(s). Person fit issues can produce misfitting items [25]. Internal consistency of the scale is calculated using the Person Separation Index (PSI) which is the ratio of true variance to observed variance using the logit scores [22]. The minimum PSI is 0.70 for group use which indicates acceptable internal consistency [22].

Examination of the fit of each item to the Rasch model is undertaken by observing the item thresholds and category probability curves. The *threshold* is the point at which there is an equal probability of the respondent selecting one option over another, in order (i.e. 2 or 3 on the item scale, not 1 or 3). RUMM2030 provides two graphical approaches for observation of the thresholds, a threshold map and the category probability curve. Disordered thresholds can exist where respondents are not selecting the responses in an ordered fashion. This can sometimes be resolved by rescoring the item in order to collapse one or more scale response options into one score. An example of this rescoring is where the original scale scoring was 1, 2, 3, 4, 5 with a disordered threshold; the item may be rescored as 1, 1, 2, 3, 4 for example. To resolve the disordering, RUMM2030 requires that scale options are coded sequentially.

Person fit issues are examined using the fit residual SD. If the SD is between -2.5 and +2.5 then the person’s response to the scale is deemed to fit the Rasch model. Generally, person’s whose responses are outside of this range are removed from the analysis.

Once any person and item issues have been resolved, differential item function (DIF) is examined. DIF is where the response to an item on the scale is consistently dependent upon a factor outside of that being measured on the scale (i.e. age, gender). In RUMM2030, DIF can be viewed graphically and in table form. In the table, a Bonferonni-adjusted statistically significant p-value indicates a significant main effect for that factor. RUMM2030 provides the opportunity to spilt items affected by DIF in order to score the item based on the factor affecting the item [25]. This may produce different subscale or total scale scores. Where DIF is undesirable, the item may need to be removed from the scale.

Residual correlations are then calculated to observe whether there is *local dependency*. Local dependency is where one item on the scale correlates with another, inflating the PSI. In RUMM2030, items that have a correlation of 0.20 or more are examined. Where there is a substantial change in the PSI (often a decrease), removal of one of the items is often required. When all scale issues have been resolved, a principal components analysis is undertaken to assess the unidimensionality of the scale. Unidimensionality is an underlying assumption of the Rasch model [22,26]. Performing a paired t-test on the items loading on the first factor (or Rasch factor) allows for the examination of whether the person estimate for the first factor differs from that of all of the items combined. When the person estimate is the same for the first factor and all scale items, the scale is determined to be *unidimensional*. Unidimensionality is a desirable outcome for scales of this type as it indicates that the scale is measuring a single underlying construct. Tennant & Conaghan [22] provide an overview of testing for dimensionality.

## Results

Two hundred and forty-five students out of 270 students enrolled in the Osteopathic Science subject completed the questionnaires, representing a 90% response rate. Descriptive statistics and Cronbach’s alpha for the DREEM subscale and total scores are presented in Table 1. Individual item scores and descriptive data are presented in the previous paper in this series.

**Table 1:** Statistics for the DREEM

Subscale	**Mean**	**Std. Deviation**	**Subscale score interpretation**	**Alpha**
Perception of teaching	34.42	6.25	‘A more positive approach’	0.870
Perception of teachers	30.69	4.25	‘Moving in the right direction’	0.703
Academic self-perception	21.08	3.86	‘Feeling more on the positive side’	0.670
Perception of atmosphere	33.15	5.37	‘A more positive atmosphere’	0.776
Social self-perception	18.03	3.47	‘Not too bad’	0.502
Total score	135.37	19.33	‘More positive than negative’	0.923

The internal consistency for the overall DREEM was well above the generally accepted level of 0.80. The Academic self-perception and Social self-perception subscales were below the minimum acceptable level of 0.70, whilst for the other three subscales (Perception of Teaching, Perception of Learning, Perception of Atmosphere) the internal consistency was acceptable.

### Overall scale

Statistics for the CFA for both models are presented in Table 2. The data from the present study did not fit either model for any of the fit statistics. The path models generated by AMOS are at Additional file 1 (Roff et al. [19] scale) and Additional file 2 (Hammond et al. [3] scale).

**Table 2:** Model statistics for the DREEM using CFA

**Statistic**	**Recommended value**	**Roff et al. model**	**Hammond et al. model**	Yusoff 5-factor, 50 item model*
χ^2^	NA	2364.65	1078.57	4475.79
χ^2^ p-value	<0.05	<0.0001	<0.0001	<0.0001
df	NA	1165	485	1165
χ^2^/df	< or = 2	2.03	2.22	3.84
Root mean square error of approximation	< or = 0.08	0.065 (CI = 0.061-0.068)	0.071 (CI = 0.65-0.76)	0.075 (No CI reported)
Goodness of fit index (GFI)	> or = 0.9	0.718	0.783	0.709
Comparative fit index (CFI)	> or = 0.9	0.694	0.731	0.724
Tucker-Lewis index (TLI)	> or = 0.9	0.678	0.707	0.710

*The model by Yusoff is presented for comparison.

The data did not fit the Rasch model as demonstrated by the statistically significant χ^2^ value (p < 0.0001). The PSI (0.922) indicated internal consistency of the DREEM. The standard deviation fit residuals for both items (1.86) and persons (1.93) were greater than 1.5 indicating that both the DREEM items and person responses did not fit the Rasch model. Poor fit residuals (>2.5) were noted for items 9, 7, 19, 27, 28 and 50, along with statistically significant χ^2^ values for items 16, 25, 27, 28 and 35, indicating a poor fit of these items to the Rasch model. Disordered thresholds were observed for items 1, 2, 5–7, 12–16, 18–24, 27, 28, 33–35, 38, 40–45, 47 and 49. Forty-four persons also failed to fit the Rasch model. Differential item functioning was analysed for each item. Age and receiving a government allowance did not impact on any items. Gender (item 45), employment (item 31) and year level (items 2, 6, 10, 15, 17, 18, 20, 22, 24, 26, 28, 31, 38, 40, 50) demonstrated DIF. Six separate Rasch analyses failed to produce a satisfactory unidimensional model fit.

### Subscale rasch analysis

As the initial Rasch analysis demonstrated multidimensionality, each of the subscales established by Roff et al. [19] were independently analysed in order to fit the Rasch model.

### Perception of teaching

The item-trait interaction was statistically significant (p = 0.000043) suggesting misfit between the data and Rasch model. The PSI was 0.853 indicating acceptable internal consistency. Person fit was acceptable (fit residual SD = 1.30) however the item fit residual SD was 1.69, beyond the recommended cut-off of 1.50.

Four separate analyses were conducted that included recoding of item response scales, and deletion of misfitting persons and items. A model fit was achieved through the deletion of 5 of 12 items (1, 7, 25, 38, 44) and the removal of data for 25 of 245 misfitting persons. No recoding of the remaining scale items was necessary. The model fit statistics were χ^2^ = 0.694, PSI = 0.819, item fit residual SD = 1.25, and person fit residual SD = 0.94. The remaining items were 13, 16, 20, 22, 24, 47, and 48 (Table 3). No threshold disordering was present and the residual correlations did not indicate any local dependency. Age, gender, employment status, and receiving a government allowance did not demonstrate DIF. Items 22 and 24 demonstrated DIF for year level. PCA demonstrated a unidimensional scale.

**Table 3:** Revised brief version of DREEM items for each Rasch analysed subscale and Rasch analysed scale

**Scale**	**Perception of Teaching**	**Perception of Teachers**	**Academic self-perception**	**Perception of Atmosphere**	**Social self-perception**	**Rasch-analysed scale**
**Items**	13, 16, 20, 22, 24, 47, 48	6, 8, 29, 32, 37, 39	10, 21, 26, 31	30, 34, 35	3, 4, 46	8, 13, 21, 22, 24, 29, 30, 32, 34, 35, 39, 48
**Hammond et al.**[3]**scale items**	1, 7, 13, 16, 20, 21, 24, 38, 44	2, 6, 8, 9, 18, 29, 39	5, 10, 26, 27, 31, 41, 45	11, 33, 34, 42	3, 14, 15, 19, 28	

Items from the DREEM developed by Hammond are presented for comparison.

### Perception of Teachers

The chi-square (p = 0.009), PSI (0.702), item fit residual (SD = 1.33) and person fit residual (SD = 1.15) demonstrated an overall fit of this subscale to the Rasch model. On further analysis, item 9 demonstrated a poor fit residual (2.898) and threshold disordering was observed for items 2, 6, 9, 18 and 40. Responses from 9 persons failed to fit the Rasch model.

Four analyses were undertaken and model fit was achieved for two of these analyses. The stronger of the two models is presented here. The model fit statistics were chi-square (p = 0.599), PSI (0.673), item fit residual (SD = 0.77) and person fit residual (SD = 0.82). Items 2, 9, 18, 40 and 50 were removed and no rescoring of the remaining items was required. Twenty-seven misfitting persons were removed from the analysis. Age, gender, employment status, and receiving a government allowance did not demonstrate DIF. Item 6 demonstrated DIF for year level. No residual correlations were identified and the PCA revealed a unidimensional scale. The 6 items in this subscale are in Table 3.

### Academic self-perception

Initial model fit was also demonstrated for the academic self-perception subscale. Fit statistics were: chi-square (p = 0.349), PSI (0.659), item fit residual (SD = 0.98) and person fit residual (SD = 1.17). No issues with the item fit residuals were identified however disordered thresholds were observed for items 5, 21, 27, 41 and 45. Twelve persons also did not fit the Rasch model.

Four Rasch analyses were undertaken to identify a model. Items 5, 27, 41 and 45 were removed. Item 21 was rescored from 0, 1, 2, 3, 4 to 0, 0, 1, 2, 3, thereby collapsing responses to the first two categories into one response. No other item rescoring was required. Three misfitting persons (out of 245) were removed. The resultant model fit statistics were: chi-square (p = 0.469), PSI (0.474), item fit residual (SD = 0.96) and person fit residual (SD = 1.05). Item 6 was affected by DIF for gender, employment and receiving a government allowance, and all items were affected by DIF for year level. No residual correlations were noted and the scale was deemed to be unidimensional based on the PCA. The remaining 4 items are listed in Table 3.

### Perception of Atmosphere

This subscale did not initially fit the Rasch model. Initial fit statistics were: chi-square (p = 0.000002), PSI (0.772), item fit residual (SD = 1.68) and person fit residual (SD = 1.26). Item 17 demonstrated poor fit to the Rasch model as evidenced by a statistically significant chi-square (p = 0.000607). Threshold disordering was observed for items 12, 23, 33, 35, 42, 43 and 49. Fourteen persons did not fit the Rasch model.

Five analyses were undertaken to identify a scale to fit the Rasch model. Items 11, 12, 17, 23, 33, 36, 42, 43 and 49 were removed along with 53 misfitting persons. The fit statistics were: chi-square (p = 0.153), PSI (0.386), item fit residual (SD = 0.83) and person fit residual (SD = 0.87). No DIF was observed for any item and no residual correlations were present. PCA indicated the scale was unidimensional with the remaining 3 items in Table 3.

### Social self-perception

Initial statistics indicated the scale fitted the Rasch model although the PSI was lower than required: chi-square (p = 0.016), PSI (0.538), item fit residual (SD = 0.96) and person fit residual (SD = 0.92). No individual item fit issues were observed however items 14, 15, 19 and 28 demonstrated disordered thresholds. Three persons did not fit the Rasch model.

Two Rasch analyses were required to produce a model. Items 14, 15, 19, 28 were removed along with 8 misfitting persons. The scale fit statistics were: chi-square (p = 0.03), PSI (0.261), item fit residual (SD = 0.86) and person fit residual (SD = 0.81). DIF by year level was identified for item 46. No residual correlations existed and the PCA demonstrated a unidimensional scale. Table 3 demonstrates the 3 items remaining for this subscale.

The results discussed above provide a Rasch analysed version of the 5-factor DREEM. Each subscale demonstrated unidimensionality after modification to fit the Rasch model however as the original 50 item DREEM did not. Subsequently, the 23 items from the subscales were combined.

### Rasch-analysed DREEM

The 23 items from the Rasch-analysed subscales were then reanalysed as one whole scale using the Rasch model, in order to determine if the modified 5-factor DREEM was unidimensional. The scale fit statistics were: chi-square (χ^2^ < 0.001), PSI (0.872), item fit residual (SD = 1.79) and person fit residual (SD = 1.51). These statistics indicate a poor fit to the Rasch model. Items 26, 46 and 50 demonstrated a poor fit and disordered thresholds were observed for items 13, 16, 20, 21, 22, 24, 34 and 47. Twenty-three misfitting persons were also identified.

**Figure 2:**
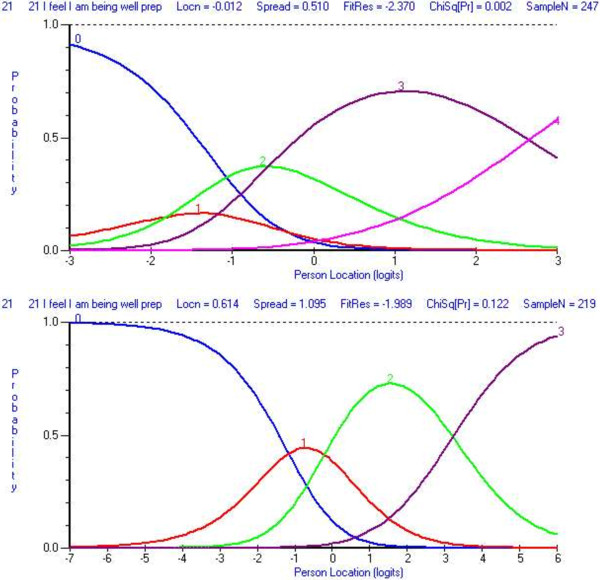
Category probably curves for item 21 (A) pre-rescore and (B) post-rescore.

**Figure 3:**
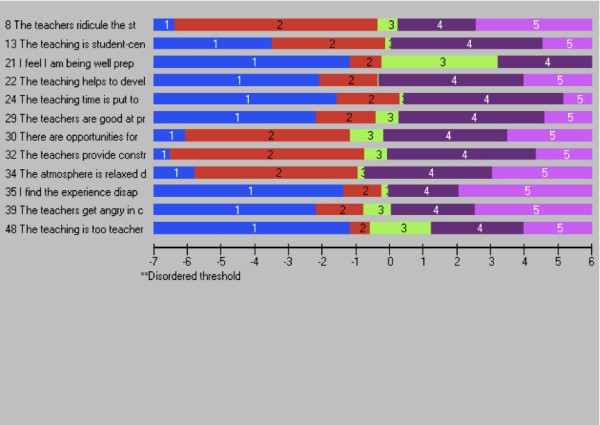
Item threshold map for the ‘short form version’ of the DREEM.

**Figure 4:**
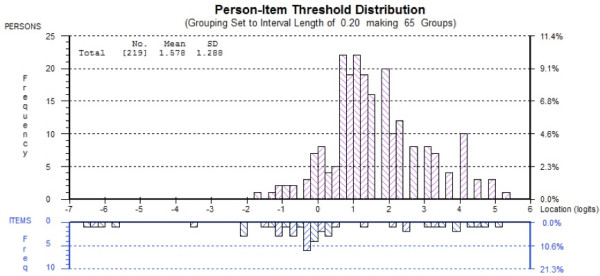
Person-item threshold distribution for the 12-item version of the DREEM.

Four Rasch models were generated. Ten items (3, 4, 10, 16, 20, 26, 31, 46, 47, 50) were deleted as were data for 28 persons. Rescoring of item 21 was required in order to resolve the threshold disordering – rather than being scored as 0, 1, 2, 3, 4 the item was scored 0, 0, 1, 2 and 3 (Figure 2). DIF for age was observed for item 37 and DIF for year level was observed at item 22, 24, and 37. Item 37 was subsequently deleted and this also resolved the DIF for item 22. The scale fit statistics following these modifications were: chi-square (χ^2^ = 0.421), PSI (0.859), item fit residual (SD = 1.04) and person fit residual (SD = 1.00). No residual correlations were observed and the PCA indicated the scale was unidimensional. The 12-item version of the DREEM can be summed to produce a total score for the scale. The threshold map for the revised scale is at Figure 3. The person-item threshold is displayed at Figure 4 and demonstrates a mean person location of 1.578.

## Discussion

This study has presented the psychometric properties of the DREEM using both classical test theory and item response theory. Data from the student population in the osteopathy program at VU did not fit either the *a*-*priori* 5-factor structure of the DREEM proposed by Roff et al. [1] nor the factor structure proposed by Hammond et al. [3]. Hammond et al. [3] noted that 17 items had fit indices less than 0.7 and should be removed. However their modelling suggested that 18 items had indices less than 0.7 and the model at Additional file 2 represents the deletion of 18 items. Significant changes to both models would be required in order to develop a model that fits the current data. Yusoff [18] demonstrated in a sample of Malaysian medical students, that a shortened version (17 items) of the DREEM was required in order to fit the original five factor structure. These results support the need for further analysis of the structure of the DREEM using item response theory, as undertaken in the present study.

Rasch analysis provides an opportunity to analyse a scale independently of the responses and can play a part in refining a scale to enhance its psychometric value. Data is fitted to the Rasch mathematical model rather than fitting the data to the a-priori structure (i.e. CFA) or factor structure identified through an exploratory factor analysis. Data from the DREEM were entered into the Rasch model and no suitable model could be identified for the full 50-item scale. Hammond et al. [3] suggested that the DREEM may be a one-factor scale. The lack of fit to the Rasch model suggests the 50-item DREEM is not unidimensional and possibly not measuring the underlying a-priori construct of *educational environment*. This result also suggests that summing the result of each item into a total score for the DREEM may be problematic and not sound practice [27].

The PSI for the 50-item version of the DREEM was 0.922 indicating that the scale is internally consistent. This is in agreement with a range of authors who have reported Cronbach’s alpha scores of 0.75 [28], 0.87 [5], 0.89 [29], 0.90 [6,30], 0.912 [31], and 0.93 [7,18,32,33]. This variability in alpha scores demonstrates the sample-dependent nature of the statistic and supports the need for authors to continue to investigate the psychometrics of the DREEM. Additionally, the alpha scores over 0.90 suggest that there may be redundancy in the DREEM items, as items that correlate strongly with each other can inflate the score.

In order to establish whether there were multiple dimensions to the DREEM, Rasch analyses were conducted for the items on each of the subscales identified by Roff et al. [19]. Rasch model fit was achieved for each of the 5 subscales with varying degrees of quality of fit. The strongest fit was the Perception of Teaching subscale where the PSI was over 0.80. This is consistent with previous research where the Cronbach’s alpha score for this subscale is often strong. For example, Hammond et. al. [3] and De Oliveira Filho et al. [34] reported alpha scores of 0.80 and 0.82 respectively in medical student populations, although Ostapczuk et al. [9] reported an alpha score of 0.70 in a German dental student population. In the current study the refinement of this subscale has not significantly impacted internal consistency and it would appear that that the remaining 7 items provide a measure of students’ perception of teaching. As a single subscale, it is unidimensional and the responses to each item can be summed to create a total score.

In the current study there were some instances of items being affected by year level in the course. Items 22 (The teaching is sufficiently concerned to develop my confidence) and 24 (The teaching time is put to good use) both demonstrated DIF for year level. These two items, along with a number of other items, exhibited different response patterns between year 2 students and all other year levels. In part 1 of this study, it was identified that there were a number of issues with the students’ perception of the osteopathy program in year 2. This difference in perception may be the reason for the presence of DIF with these items, and future studies should investigate whether student year level affects these items.

There were a number of issues identified that related to the items themselves and the way that participants completing the measure responded to them. It is possible that the issue may lie in the wording of the item, or the use of a ‘neutral’ response category. Negatively worded or phrased items are potentially problematic [35] although they are used to avoid systematic response bias issues. The phraseology can mean that the interpretation of the item varies from person to person in a non-uniform manner and can impact on the psychometrics of a scale, both in CTT and IRT. In the present study, a number of negatively phrased items were retained in the brief version of the measure. An example is item 11 (The teaching is too teacher-centred) in the Perception of Teaching subscale. Conversely, item 9 (The teachers are authoritarian) was removed. Students possibly perceived this to be quite a strong statement and responding to the item in such a way that it did not fit the expected score for that item.

It has been previously suggested there may be issues with neutral response options in self-report measures and some have counselled against this method of scoring [36,37]. Kulas et al. [38] have suggested this is particularly so if the respondents do not perceive the response category to be a true mid-point of the statements on the scale. These authors suggested the term ‘dumping ground’ for this type of midpoint as respondents are likely to use it when they are unsure of how to respond to the item. In the current study it is possible that a number of items that were removed during the Rasch analysis because it was not possible to rescore the items. Responses can only be collapsed together to rescore the item where they are similar with another adjacent response option [37]. For example, in the DREEM the ‘strongly disagree’ and ‘disagree’ responses can be collapsed together, as they both represent the same *do not agree* responses. It is not possible to collapse another response with the neutral response category. Item 21 in the present study provides an example of this rescoring and the effect is demonstrated at Figure 2.

### Five-factor DREEM

The present study has developed a modified version of the DREEM based on individual Rasch analyses of the 5 subscales (Table 3). Each subscale was analysed to determine if the items were measuring the construct of each factor. Fit to the Rasch model was achieved for all 5 subscales albeit that the PSI for 4 out the 5 subscales was below an acceptable level of 0.70 [22]. Only the Perception of Teaching subscale achieved an acceptable PSI. The authors suggest caution should be applied if the original version of the DREEM (50 items) is to be used, given the low PSI values for the majority of the subscales. These low PSI values highlight the difference between the sample-dependent nature of Cronbach’s alpha and the sample-independent PSI. If subsequent studies are to use this 5-factor version of the DREEM, a total score should not be generated, given that it consists of 5 independent unidimensional scales. However, the items within each subscale can be summed. As in the present study, the studies by Hammond et al. [3] and Yusoff [18] removed a substantial number of items from the DREEM in order to develop a psychometrically sound scale.

It is important to review the items that have been removed as they may investigate important aspects of the educational climate, and could be reworded; or the scoring adapted for all items, to remove the neutral response category, or collapsed with other similar items. Although negatively worded items have been reported to be problematic, there are instances in the Rasch analysed subscales that are still worded in this manner.

### ‘Short-form’ DREEM

Model fit was achieved for a 12-item scale that was unidimensional. Item 24 (The teaching time is put to good use) demonstrated DIF for age with those between 18–20 years of age. There is very little overlap with the 17-item scale developed by Yusoff [18] with only items 22, 24 and 30 appearing in both the short-form version of the DREEM developed in the present study and that developed by Yusoff. A possible reason for this difference is the use of CTT in the Yusoff [18] study and the use of IRT in the present study. Further work to validate the 17-item in the Yusoff study is required. The 12-item version of the DREEM developed in the present study could potentially be used as a ‘short-form’ version given that it contains items from each of the original DREEM subscales, except Social self-perception. The advantage of such a scale is the potential acceptability to respondents and efficiency of administration. This revised version of the DREEM can be summed to produce a total score for the entire scale given its unidimensional nature [39]. Validation of the short-form version is required to evaluate the learning environment prior to using it as a valid or reliable measure with health science student populations. Preferably this should be undertaken with additional student samples, through comparison with responses to the full DREEM [26].

In future studies the authors will investigate the test-retest reliability and concurrent validity of the DREEM and the revised 12-item measure, as well as conducting further investigations of the factor structure. The results obtained in the current study cannot be over generalised but they do pose some interesting questions regarding the reliability of the full version of the DREEM measure for use with health science students in Australia. Some considerations are that this study was based on a single administration of the DREEM in one allied health program (osteopathy) in Australia. The results obtained may not be generalisable to other health professional education programs either in Australia or internationally. As such, other authors are encouraged to replicate the present study, particularly the Rasch analysis, with their own data in order to continue developing and strengthening the DREEM.

## Conclusions

The DREEM is a widely used scale to measure the educational environment in health professional education programs. Previous studies have questioned the psychometric properties of the measure. The present study employed both CTT and IRT to investigate and develop a psychometrically sound version of the DREEM. Two versions of the DREEM were produced with the Rasch analysis. One version is based on the 5-factor scale proposed by the original authors and the other a 12-item ‘short-form’ version. The 5-factor version requires further testing, given the issues identified with the poor internal consistency of four subscales. The ‘short-form’ version requires validation and reliability testing. Those institutions that use the DREEM are encouraged to investigate and report the psychometric properties of the measure in their own populations. Caution is also advised when calculating a total score the 50-item DREEM given the results of the present study suggest that it is not measuring the single underlying construct of educational environment.

## Competing interests

The authors declare that they have no competing interests.

## Authors’ contributions

BV designed the study. BV, JM and PMcL undertook the literature review, statistical analysis and manuscript development. All authors approved the final version of the manuscript.

## Authors’ information

Brett Vaughan is a lecturer in the College of Health & Biomedicine, Victoria University, Melbourne, Australia and a Professional Fellow in the School of Health & Human Sciences at Southern Cross University, Lismore, New South Wales, Australia. His interests centre on competency and fitness-to-practice assessments, and clinical education in allied health.

Jane Mulchay is a lecturer in the College of Health & Biomedicine at Victoria University, Melbourne, Australia. Her interests include scale development and population health.

Patrick McLaughlin is a senior lecturer in the College of Health & Biomedicine at Victoria University, Melbourne, Australia. His interests include research methods, statistics and biomechanics.

## Pre-publication history

The pre-publication history for this paper can be accessed here:

http://www.biomedcentral.com/1472-6920/14/100/prepub

## Supplementary Material

Additional file 1 CFA path model for the Roff et al. scale. Click here for file

Additional file 2 CFA path model for the Hammond et al. scale. Click here for file
