# Supplementary figures and images for: The DREEM, part 2: psychometric properties in an osteopathic student population
Source: BMC Med Educ. 2014 May 20;14:100. doi: 10.1186/1472-6920-14-100 (PMC4050100; doi:10.1186/1472-6920-14-100)

### Additional file 1 – CFA path model for the Roff et al. scale

###
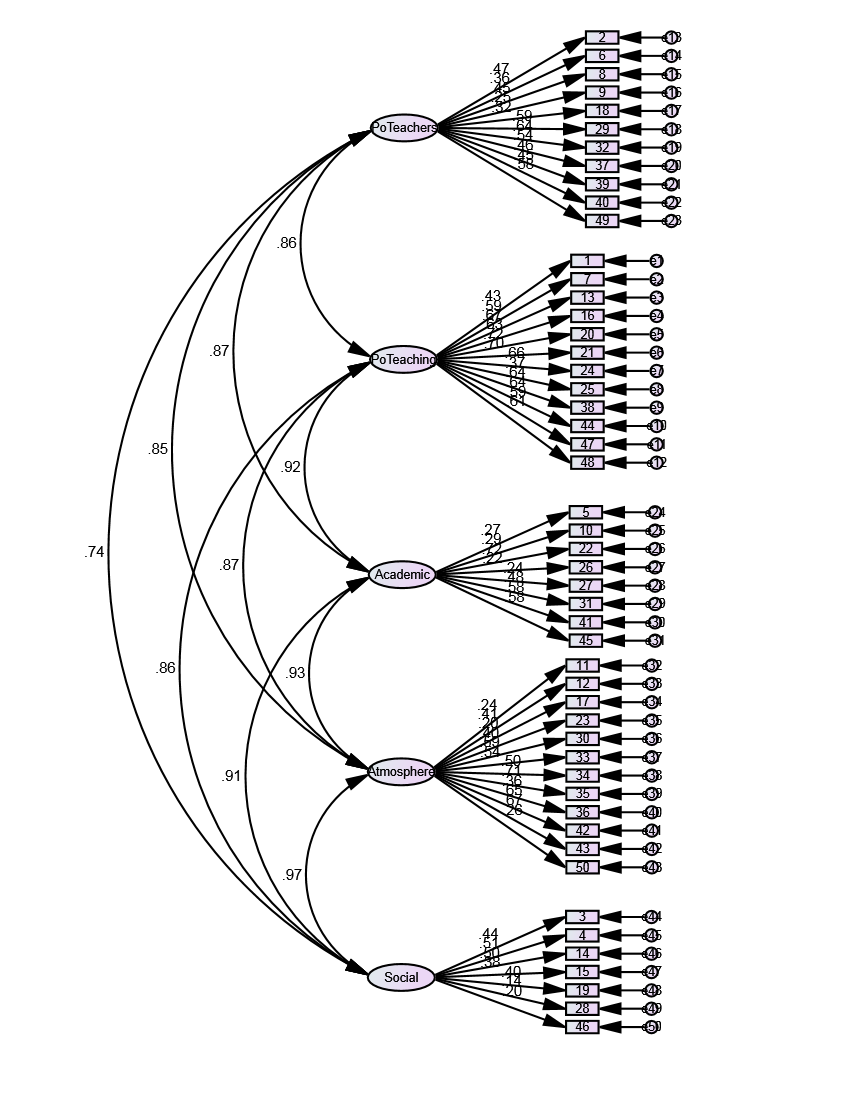

Supplement: Additional file 1 — CFA path model for the Roff et al. scale. [file 1472-6920-14-100-S1.doc]

### Additional file 2 – CFA path model for the Hammond et al. scale


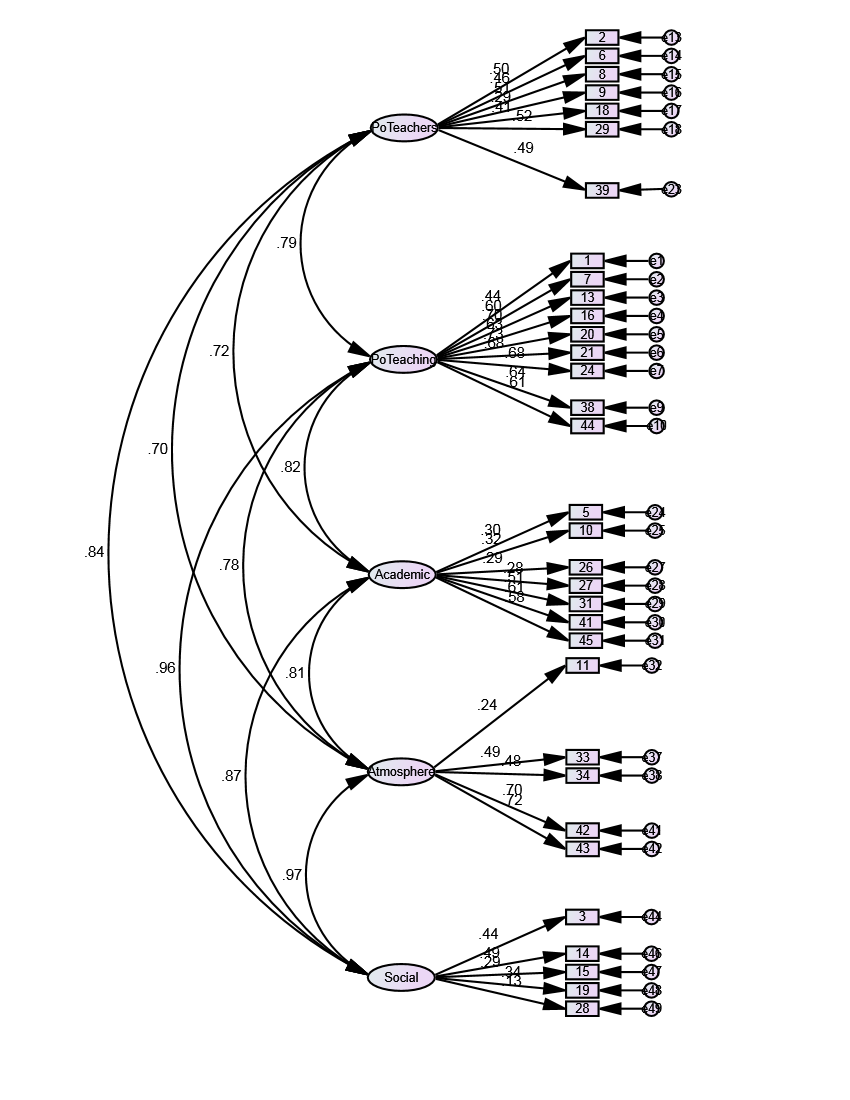

Supplement: Additional file 2 — CFA path model for the Hammond et al. scale. [file 1472-6920-14-100-S2.doc]
